# Deep learning-based multi-functional therapeutic peptides prediction with a multi-label focal dice loss function

**DOI:** 10.1093/bioinformatics/btad334

**Published:** 2023-05-22

**Authors:** Henghui Fan, Wenhui Yan, Lihua Wang, Jie Liu, Yannan Bin, Junfeng Xia

**Affiliations:** Information Materials and Intelligent Sensing Laboratory of Anhui Province and Key Laboratory of Intelligent Computing and Signal Processing of Ministry of Education, Institutes of Physical Science and Information Technology, Anhui University, Hefei, Anhui 230601, China; Information Materials and Intelligent Sensing Laboratory of Anhui Province and Key Laboratory of Intelligent Computing and Signal Processing of Ministry of Education, Institutes of Physical Science and Information Technology, Anhui University, Hefei, Anhui 230601, China; Information Materials and Intelligent Sensing Laboratory of Anhui Province and Key Laboratory of Intelligent Computing and Signal Processing of Ministry of Education, Institutes of Physical Science and Information Technology, Anhui University, Hefei, Anhui 230601, China; Information Materials and Intelligent Sensing Laboratory of Anhui Province and Key Laboratory of Intelligent Computing and Signal Processing of Ministry of Education, Institutes of Physical Science and Information Technology, Anhui University, Hefei, Anhui 230601, China; Information Materials and Intelligent Sensing Laboratory of Anhui Province and Key Laboratory of Intelligent Computing and Signal Processing of Ministry of Education, Institutes of Physical Science and Information Technology, Anhui University, Hefei, Anhui 230601, China; Information Materials and Intelligent Sensing Laboratory of Anhui Province and Key Laboratory of Intelligent Computing and Signal Processing of Ministry of Education, Institutes of Physical Science and Information Technology, Anhui University, Hefei, Anhui 230601, China

## Abstract

**Motivation:**

With the great number of peptide sequences produced in the postgenomic era, it is highly desirable to identify the various functions of therapeutic peptides quickly. Furthermore, it is a great challenge to predict accurate multi-functional therapeutic peptides (MFTP) via sequence-based computational tools.

**Results:**

Here, we propose a novel multi-label-based method, named ETFC, to predict 21 categories of therapeutic peptides. The method utilizes a deep learning-based model architecture, which consists of four blocks: embedding, text convolutional neural network, feed-forward network, and classification blocks. This method also adopts an imbalanced learning strategy with a novel multi-label focal dice loss function. multi-label focal dice loss is applied in the ETFC method to solve the inherent imbalance problem in the multi-label dataset and achieve competitive performance. The experimental results state that the ETFC method is significantly better than the existing methods for MFTP prediction. With the established framework, we use the teacher–student-based knowledge distillation to obtain the attention weight from the self-attention mechanism in the MFTP prediction and quantify their contributions toward each of the investigated activities.

**Availability and implementation:**

The source code and dataset are available via: https://github.com/xialab-ahu/ETFC.

## 1 Introduction

The therapeutic peptides are short amino acid (AA) monomer chains with sizes ranging from 5 AA to 50 AA in length that play important roles as anti-infectives, hormones, biological messengers, and neurotransmitters (Basith *et al.* 2020). In recent years, with the development of high-throughput sequencing technologies and experimental data acquisition techniques, more and more multi-functional therapeutic peptides (MFTP) have been identified (Wei *et al.* 2019, Xiao *et al.* 2021, Tang *et al.* 2022). For example, some host-defense peptides from frog skin have more than one therapeutic property, including antiviral, antidiabetic, anticancer, and immunomodulation properties (Conlon *et al.* 2014). In addition, because of the crucial advantages of high specificity and selectivity, reduced toxicity, and short half-life (Vlieghe *et al.* 2010, Marqus *et al.* 2017), therapeutic peptides are safer than traditional drugs. MFTP-based therapeutics may be excellent candidates for developing novel peptide drugs for the treatment of various diseases.

In the postgenomic era, there is an enormous amount of peptide sequences with unknown functions (Bateman *et al.* 2021). The wet laboratory experiments used to identify functional peptides are time-consuming and laborious and severely affect development efficiency. How to efficiently and accurately explore more functions of these peptides has become one of the most pressing challenges. Sequence-based computational methods offer a highly efficient way of predicting the functional therapeutic peptides on a large scale and have been proposed as a primary means of screening for biologists (Bin *et al.* 2020, Dai *et al.* 2021, Chen *et al.* 2022, Zhang *et al.* 2022).

multi-label classification (MLC) based on machine learning algorithms is a highly desirable method to identify MFTP. Problem transformation and algorithm adaptation are the two most common approaches for MLC (Tarekegn *et al.* 2021). The problem transformation approach transforms MLC into a set of independent binary classifications (Wei *et al.* 2019, Zhang and Zou 2020, Guo *et al.* 2021). This approach is widely applied and fundamental to many MLC tasks but neglects the label correlation among the binary classifications. The algorithm adaptation approach, considers the label correlation to mitigate the drawbacks of binary classifications (Tarekegn *et al.* 2021). For example, Xiao et al. used algorithm adaptation-based MLC to predict the functional activities of the antimicrobial peptides (AMP) (Xiao *et al.* 2013, Xiao *et al.* 2021). In 2022, our team proposed a DNN-based MLC method MLBP to predict five classes of bioactive peptides (Tang *et al.* 2022). In the same year, our team improved another MLC method PrMFTP based on DNN and multihead self-attention mechanism (MHSA) for the identification of MFTP with 21 classes of functions (Yan *et al.* 2022). Although computational methods have been developed and achieved success in MFTP prediction, algorithm improvement and higher latent feature representations could promote the predicted performance.

Furthermore, these existing methods all utilized imbalanced datasets, where the number of peptides from the minority classes is exceedingly fewer than those from the majority classes. And the data imbalance problem limits the performance of large-scale high-throughput prediction. To handle the data imbalance problem, some works resampled data to produce a new, balanced set (Liu *et al.* 2008, Lin and Xu 2016, Castellanos *et al.* 2018). PrMFTP, our previous work, used the class weight optimization method to address the imbalanced problem and achieved a great improvement in the prediction performance (Yan *et al.* 2022). Some of the other cross-entropy loss (CEL)-based methods have achieved success in dealing with imbalanced datasets. For example, Lin *et al.* (2017) proposed the focal loss (FL) that could focus on hard-to-classify samples in imbalanced datasets. Ridnik *et al.* (2021) proposed FL-based asymmetric loss (ASL) for MLC. However, these CEL-based methods only calculate the loss between the prediction probability and label of each class in MLC. They neglect the association among these classes.

To handle the above problems, we present ETFC, a novel DNN-based MLC method for predicting MFTP. This work can be summarized as follows: (i) In the ETFC model, semantic-based and position-based embedding block combined with MHSA can capture more peptide sequence information, and text convolutional neural network (TextCNN) could extract the more effective information from peptide sequence. (ii) To handle the imbalance problem in the MLC dataset, we design a novel loss function, termed multi-label focal dice loss (MLFDL), for MLC based on FL and dice loss (DL). MLFDL can dynamically assign weights to labels by exploiting label correlations to improve the prediction performance. (iii) We use the teacher–student-based knowledge distillation (KD) to obtain the importance of AA and quantify their contributions toward each of the investigated activities.

## 2 Materials and methods

### 2.1 Materials

In this study, we use the multi-label benchmark MFTP dataset (Yan *et al.* 2022), which contains 9841 therapeutic peptides, categorized into 21 classes: antiangiogenic peptide, antibacterial peptide (ABP), anticancer peptide (ACP), anticoronavirus peptide, antidiabetic peptide (ADP), antiendotoxin peptide, antifungal peptide (AFP), anti-HIV peptide, antihypertensive peptide (AHP), anti-inflammatory peptide, anti-MRSA peptide, antiparasitic peptide, antitubercular peptide (ATP), antiviral peptide (AVP), blood–brain barrier peptide, biofilm-inhibitory peptide, cell-penetrating peptide, dipeptidyl peptidase IV peptide (DPPIP), quorum-sensing peptide, surface binding peptide, and tumor homing peptide. In all, 80% of the benchmark dataset forms the training set, which is utilized for model construction and hyperparameter optimization. The rest 20% is the test set, which is applied for the model evaluation. Note that the evaluation of MultiPep (Grønning *et al.* 2021) is performed on another test set, which consists of the eight shared classes (ABP, ACP, ADP, AFP, AHP, ATP, AVP, and DPPIP) of peptides between ETFC and MultiPep.

### 2.2 Method framework

**Figure 1:**
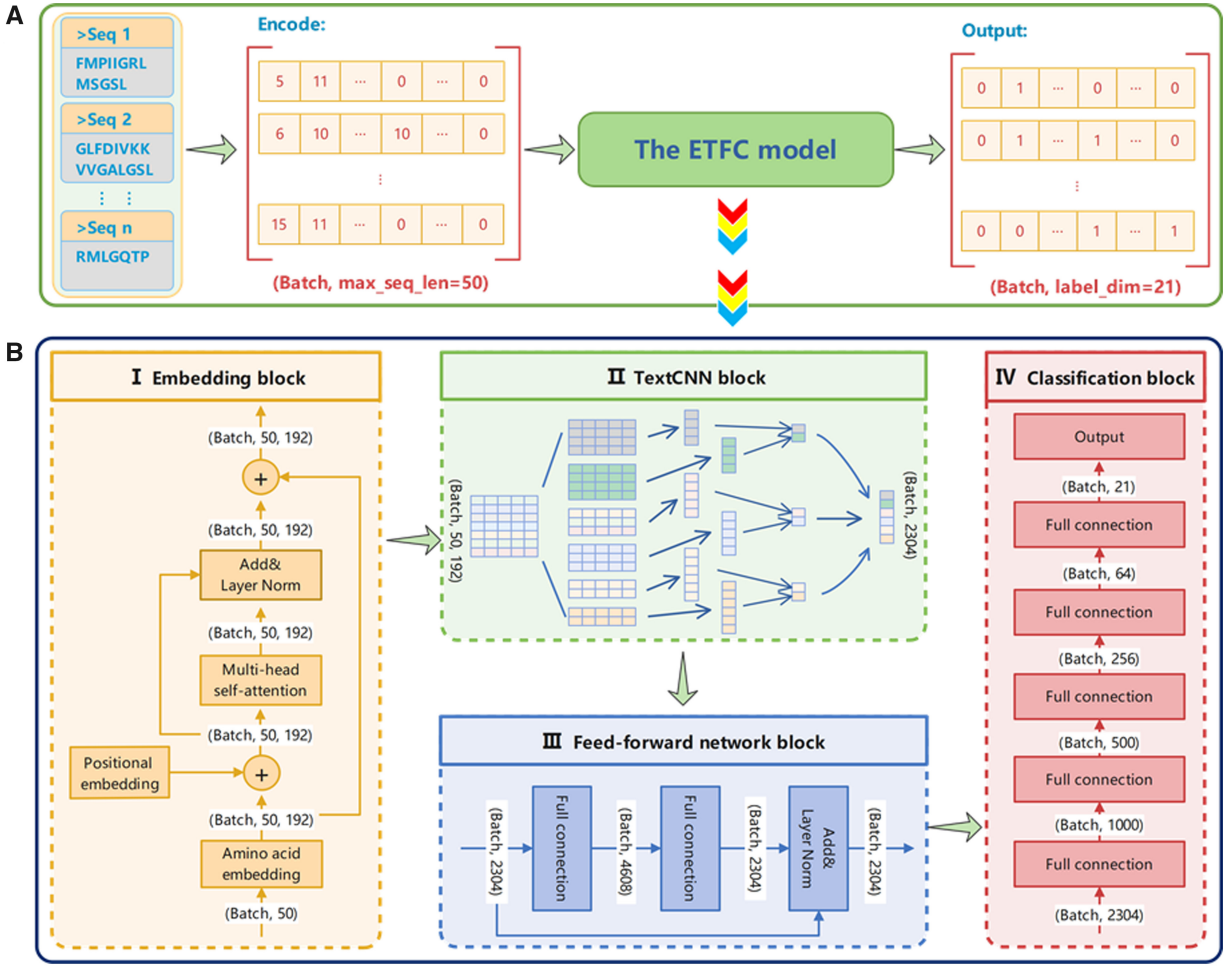
The framework of the ETFC method for MFTP prediction. (A) The overall workflow schema of the ETFC method for MFTP prediction. (B) The overview of the proposed ETFC model architecture, which composes of four blocks: (I) embedding block, (II) TextCNN block, (III) feed-forward network block, and (IV) classification block. Max_seq_len = 50, the maximum sequence length is 50. Label_dim = 21, the dimension of the label vector is 21. The embedding dimension is 192

Figure 1a shows the framework of the ETFC method for MFTP prediction with three main steps. First, the peptide sequence is encoded into the sequence coding matrix. According to the alphabetical order of 20 native AAs’ single-character codes (A, C, D, E, F, G, H, I, K, L, M, N, P, Q, R, S, T, V, W, and Y), we use the numerical codes 1, 2, 3, …, 20 to denote these AAs in the peptide sequences. In the benchmark dataset, the length of peptide sequences ranges from 5 AA to 50 AA. To ensure the numerical inputs have the same dimension, the sequences of length <50 AA are padded with 0–50 AA. Second, the sequence coding matrix is imported into the ETFC model architecture (embedding block + TextCNN block + feed-forward network block + classification block) to extract features and perform classification (Fig. 1b). Finally, with the sigmoid activation function, the probability scores of each sequence to be the 21 categories of therapeutic peptides are produced. These probability scores are converted into a 21-dimension prediction vector with cutoff threshold = 0.5, which corresponds to the prediction labels for each category. The details of the threshold selection are shown in Supplementary Table S1. In addition, we train the model with MLFDL, which is an imbalanced learning strategy. The details of the framework are shown subsequently.

#### 2.2.1 Model architecture

Figure 1b shows the overview of the proposed ETFC model architecture with four main blocks: embedding block, TextCNN block, feed-forward network block, and classification block. The details of these blocks are described as follows.

- Embedding block
- Through numerical coding and filling, we obtain the sequence coding matrix of peptide sequences with the same length (50 AA). In the embedding block (Fig. 1b and Supplementary Fig. S1), an embedding matrix containing AA semantic information and AA position information is generated. First, the sequence coding matrix is converted into semantic embedding matrix *X* through the embedding method with the AA semantic information. The semantic embedding matrix *X* is a *d*-dimensional vector representation of peptide sequence with length *n* ($X\in R^{n\times d}$, *n *=* *50, *d *=* *192). Second, we adopt the positional embedding method (Vaswani *et al.* 2017, Chu *et al.* 2022) to extract the absolute position information of each AA in the peptide sequence, and obtained the position embedding matrix *P*, which is represented by the following equations:
  (1) $$p_{i,2j}=\sin\left(\frac{i}{{10\;000}^{2j/d}}\right),$$
  (2) $$p_{i,2j+1}=\cos\left(\frac{i}{{10\;000}^{2j/d}}\right),$$
  where *i* is the *i*th AA in the peptide sequence, *j* is the *j*th dimension, and *d* is the dimension of the position embedding matrix. More precisely, each dimension of the position embedding corresponds to a sinusoid or cosinusoid. Then, we use the MHSA (Lin *et al.* 2017) to further extract features of the embedding matrix, hoping to obtain more AA semantic information and AA relative position information. Finally, we obtain an embedding matrix that not only contains the semantic information of the AAs in the sequence but also covers the positional information of the AAs.
- TextCNN block
  In this block, as a kind of multiscale CNN, TextCNN (Zhang and Wallace 2015) uses different sizes of convolution kernels to convolve the embedding matrix representation and extract the associated information between AAs with various distances. Since the minimum length of peptide sequence in the benchmark dataset is 5 AA, the sizes of convolution kernels are set to be 2, 3, 4, and 5. After the feature vectors are obtained by convolution, we use the maximum pooling layer to reduce dimension. Ultimately, we obtain the pooled feature representation, which covers the global feature representation of the peptide sequence.
- Feed-forward network block
  In the feed-forward network block, we import the features into a multilayer perception to enhance the feature representation (Vaswani *et al.* 2017, Chu *et al.* 2022). The multilayer perception is composed of two full connection layers. The residual connection and layer normalization are used to prevent the vanishing gradient problem in gradient backpropagation. The feature input to the block is defined as $Y\in R^{m\times k}$, m represents the number of peptide sequences, and k represents the dimension of feature representation for each peptide sequence. The mathematical description of the block is as follows:
  (3) $$\mathrm{FOB}\left(Y\right)=LN\left(\max\left(0,Yw_{1}+b_{1}\right)w_{2}+b_{2}+Y\right),$$
  where $w_{1}$ and $b_{1}$ are the weights and offsets on the first fully connected layer, respectively, $w_{2}$ and $b_{2}$ are the weights and offsets on the second fully connected layer, respectively, and *LN* represents layer normalization.
- Classification block
  The classification block is composed of full connection layers and is employed to reduce the dimension of the feature representation vector for each peptide sequence. And the prediction results of the model are mapped between 0 and 1 by a sigmoid function. Finally, we obtain the prediction probability score on various functions for peptide sequences.

#### 2.2.2 Multi-label focal dice loss

In the MLC task, for the *m*-th label of the *n*-th sample $y_{n}^{m}$, the prediction probability $p_{n}^{m}$ is defined as the foreground probability and$\;(1-p_{n}^{m})$ is defined as the background probability. $p_{n}^{m}$ represents the probability of the peptide sequence having the *m*th label. $\left(1-p_{n}^{m}\right)$ represents the probability of the peptide sequence not having the *m*th label.

Being inspired by FL, we add the modulating factors$\;[\min(p_{n}^{m}+\varepsilon_{1},\;1)]$ and$\;\left[1-\max\left(p_{n}^{m}-\varepsilon_{0},\;0\right)\right]\;$to DL. $\varepsilon_{1}$ and $\varepsilon_{0}\in [0,\;1]$ are the tunable focal factors for the foreground and background probability, respectively. The relevant mathematical expressions are as follows: where $p_{n}^{m1}$ and $p_{n}^{m0}$, respectively, represent the foreground probability and the background probability of the *m*th label of the *n*th sample. Taking Equation (4) as an example, if $p_{n}^{m}\leq 1-\varepsilon_{1}$, it is found that $p_{n}^{m1}=(p_{n}^{m}+\varepsilon_{1})p_{n}^{m}$, and $p_{n}^{m1}\leq p_{n}^{m}$, which increases its weight proportion in the loss function. Through such processing, we can dynamically adjust the prediction probability values of sample labels and make the loss function pay more attention to sample labels that are misclassified or difficult to classify.


(4) $$p_{n}^{m1}=[\min(p_{n}^{m}+\varepsilon_{1},\;1)]p_{n}^{m},$$

(5) $$p_{n}^{m0}=[1-\max(p_{n}^{m}-\varepsilon_{0},\;0)](1-p_{n}^{m}),$$

In this work, based on FL (Lin et al. 2017) and DL (Li *et al.* 2019), we use $p_{n}^{m1}$ and $p_{n}^{m0}$ to develop a novel loss function MLFDL. MLFDL can address the inherent imbalance problem in multi-label datasets. This function takes values between 0 and 1, and we aim to minimize it. MLFDL is represented as follows: where ω∈[0, 1] is a balance factor to balance foreground loss $L_{n1}$ and background loss $L_{n0}$. A simple example of calculation procedures for the molecular parts of MLFDL is shown in Supplementary Fig. S2. From Equations (6–8), we find that in DL-based MLFDL, the prediction loss of each sample is not only related to the prediction probability of a certain label, but also the prediction probabilities of other labels. To better understand this loss function, the gradient of MLFDL with respect to $p_{i}^{j}$ is as follows:


(6) $$L_{n1}=\left(1-\frac{2\sum_{m=1}^{M}p_{n}^{m1}y_{n}^{m}}{\sum_{m=1}^{M}{\left(p_{n}^{m1}\right)}^{2}+\sum_{m=1}^{M}{\left(y_{n}^{m}\right)}^{2}}\right),$$

(7) $$L_{n0}=\left(1-\frac{2\sum_{m=1}^{M}p_{n}^{m0}\left(1-y_{n}^{m}\right)}{\sum_{m=1}^{M}{\left(p_{n}^{m0}\right)}^{2}+\sum_{m=1}^{M}{\left(1-y_{n}^{m}\right)}^{2}}\right),$$

(8) $$\mathrm{MLFDL}=\sum_{n=1}^{N}\left[\omega L_{n1}+(1-\omega )L_{n0}\right],$$

(9) $$\frac{\partial L_{i1}}{\partial p_{i}^{j1}}=\left[\frac{-2y_{i}^{j}\left(\sum_{m=1}^{M}{\left(p_{i}^{m1}\right)}^{2}+\sum_{m=1}^{M}{\left(y_{i}^{m1}\right)}^{2}\right)+4p_{i}^{j1}\sum_{m=1}^{M}p_{i}^{m1}y_{i}^{m}}{{\left[\sum_{m=1}^{M}{\left(p_{i}^{m1}\right)}^{2}+\sum_{m=1}^{M}{\left(y_{i}^{m1}\right)}^{2}\right]}^{2}}\right].$$

(10) $$\frac{\partial L_{i0}}{\partial p_{i}^{j0}}=\left[\frac{-2\left(1-y_{i}^{j}\right)\left(\sum_{m=1}^{M}{\left(p_{i}^{m0}\right)}^{2}+\sum_{m=1}^{M}{\left(1-y_{i}^{m0}\right)}^{2}\right)+4p_{i}^{j0}\sum_{m=1}^{M}p_{i}^{m0}\left(1-y_{i}^{m}\right)}{{\left[\sum_{m=1}^{M}{\left(p_{i}^{m0}\right)}^{2}+\sum_{m=1}^{M}{\left(1-y_{i}^{m}\right)}^{2}\right]}^{2}}\right].$$

(11) ∂pij1∂pij=2pij+ε1, 1,  if pij<1-ε1 otherwise .

(12) ∂pij0∂pij=2pij-2-ε0, -1,  if pij>ε0 otherwise.

(13) $$\begin{array}{c} \frac{\partial \mathrm{MLFDL}}{\partial p_{i}^{j}}=\omega \frac{\partial L_{i1}}{\partial p_{i}^{j}}+\left(1-\omega \right)\frac{\partial L_{i0}}{\partial p_{i}^{j}}\\ =\omega \frac{\partial L_{i1}}{\partial p_{i}^{j1}}\cdot \frac{\partial p_{i}^{j1}}{\partial p_{i}^{j}}+\left(1-\omega \right)\frac{\partial L_{i0}}{\partial p_{i}^{j0}}\cdot \frac{\partial p_{i}^{j0}}{\partial p_{i}^{j}} \end{array}.$$

From Equations (9–13), we can see that when calculating the gradient of the probability of a certain prediction label, the gradient value is not only related to the probability of the prediction label, but also the probability of other prediction labels. In other words, MLFDL takes into account the correlation between labels both in the calculation of sample losses and in the process of gradient backpropagation.

Overall, by introducing the modulating factors and the correlation among labels, MLFDL can focus and strengthen the prediction attention on the misclassified or difficult-to-classify sample labels.

### 2.3 Knowledge distillation-based interpretation

**Figure 2:**
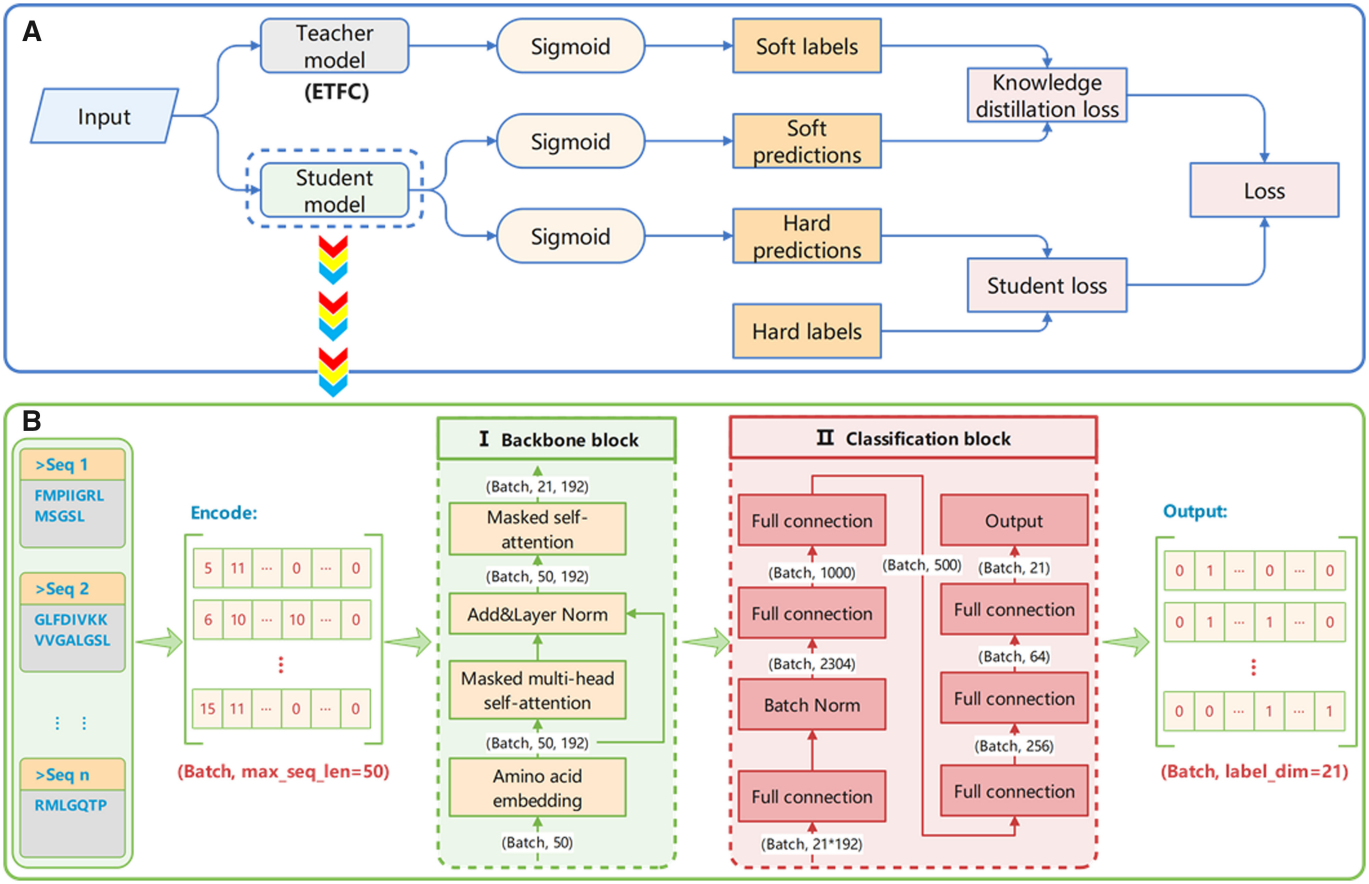
The teacher–student framework for KD. (a) The workflow of KD. ETFC is the teacher model. The student model is proposed to increase the interpretability of ETFC. (b) The overview of the student model is composed of two main blocks: the backbone block and the classification block. Max_seq_len, the maximum sequence length; Label_dim, the dimension of the label vector

KD (Hinton *et al.* 2015) is used to transfer the dark knowledge learned by a teacher model to a student model. The teacher model is usually more sophisticated and larger than the student model. Dark knowledge is the important information hidden in the predicted probabilities for all classes. In this work, ETFC achieves quite competitive performance on predicting MFTP. However, as a cumbersome method, ETFC lacks decision-making transparency (Zhang *et al.* 2018). Based on previous research (Ding *et al.* 2018, Chen *et al.* 2019), we use the teacher–student architecture-based KD to investigate the contribution importance of each AA in the ETFC for each peptide sequence. Figure 2 shows the teacher–student workflow for KD and the student model.

As illustrated in Fig. 2a, the teacher model is our proposed ETFC model, and the student model mainly consists of two parts: the backbone and classification blocks (Fig. 2b). The backbone block is used to extract the correlation information between the AAs in the peptide sequence, and transform the representation of the AA feature vector by masked self-attention mechanism. And the classification block is used to obtain the predicted score of the model. The details of the student model are exhibited in Supplementary Methods 1.1.

In the KD process, the student model mimics the teacher model with the same input. The outputs of the teacher and student models are all activated by the sigmoid function. The predicted score vector *t* of the teacher model is generated as follows: where $q_{n}$ is the *n*th input sequence and $t_{n}^{m}(q_{n})$ is the output score of the model for the *m*th label of the *n*th peptide sequence. Then, the scores could be converted into probabilities $T_{n}^{m}$:


(14) $$t_{n}(q_{n})=\left[t_{n}^{1}(q_{n}),\;t_{n}^{2}(q_{n}),\;\ldots ,\;t_{n}^{m}(q_{n})\right],$$

(15) $$T_{n}^{m}=\frac{1}{1+e^{-t_{n}^{m}(q_{n})}},$$

Similar to that of the teacher model, the output probability of the student model is represented as $S_{n}^{m}$. In the KD framework, we train the student model with real sample labels as hard labels and enable the representation learning of the student model by optimizing MLFDL, and we use the student loss (SL) to represent the loss of this process: where $p_{m1}$ and $p_{m0}$ are the probability sets obtained from Equations (4 and 5), respectively, and *y* is the set of sample labels. Here, we ignore the index of samples and labels for ease of expression.


(16) $$SL=\omega \left[1-\frac{2p_{m1}y}{p_{m1}^{2}+y^{2}}\right]+(1-\omega )\left[1-\frac{2p_{m0}(1-y)}{p_{m0}^{2}+{\left(1-y\right)}^{2}}\right],$$

Hard labels typically provide information about predicted classes, while soft labels can deliver more informative and salient knowledge from the probability distribution of all classes. To facilitate knowledge transfer from a teacher model to a student model, we employ the CEL. And we use the knowledge distillation loss (KDL) to represent the process: where $T_{n}^{m}$ and $S_{n}^{m}$ represent the prediction probability of the *m*th label of the *n*th sequence in the teacher and student models, respectively. *N* is the number of samples, and *M* represents the number of labels for each sample.


(17) $$\mathrm{KDL}=-\sum_{n=1}^{N}\sum_{m=1}^{M}\left[T_{n}^{m}\;\text{log}\;\left(S_{n}^{m}\right)+(1-T_{n}^{m})\log\left(1-S_{n}^{m}\right)\right]\;,$$

By combining SL and KDL, the total loss (TL) can be expressed as follows: where the parameter μ balances the information between the teacher and the student models.


(18) TL=SL+μ·KDL,

### 2.4 Performance metrics

To evaluate the performance of our proposed method, five commonly used metrics, are used in this MLC task. They are Precision, Coverage, Accuracy, Absolute true, and Absolute false. Among the five performance measurements, Accuracy and Absolute true are the two most significant metrics. The calculation of these metrics is as follows: where *N* is the total number of peptide sequences in the dataset, *M* is the total number of label types in the label set, $L_{i}$ represents the true label set of the *i*th sample, $L_{i}^{*}$ represents the predicted label set of the *i*th sample and


(19) Precision=1N∑i=1NLi∩Li*Li*Coverage=1N∑i=1NLi∩Li*LiAccuracy=1N∑i=1NLi∩Li*Li∪Li*Absolute true=1N∑i=1NΔ(Li, Li*)Absolute false=∑i=1NLi∪Li*-Li∩Li*M,

(20) Δ(Li, Li*)= 1, if Li* is identical to Li0, other .

### 2.5 Implementation details

In this work, we use the PyTorch framework to build our prediction model. To find the optimal hyperparameters of the model, we use the grid search optimization method. Note that learning rate optimization is performed by the tree-structured Parzen estimator approach (Bergstra *et al.* 2011). In the process of hyperparameters optimization, the various sets of hyperparameters are tested on the training dataset using 5-fold cross-validation. This process involves splitting the training data into five equally sized parts, with one part retained for validation and the remaining four used for training. The data split is repeated five times, and the mean of Absolute true is taken into account to determine the optimal hyperparameter set. To improve the reproducibility and reliability of our model, we provide the values of each hyperparameter in Supplementary Table S2.

## 3 Results and discussion

### 3.1 MLC models based on different DNN

For predicting MFTP, MHSA can extract and optimize the correlation information between AAs at any distance in the sequence, and TextCNN can extract sequence information at different distances to achieve local and global features. We use MHSA and TextCNN as the base models and proposed five different MLC models. These models are trained with MLFDL. The performance of the different models on the training and test sets is shown in Table 1.

**Table 1.** The performance of MLCs models for MFTP prediction.^a^

Model	Dataset	Precision ↑	Coverage ↑	Accuracy ↑	Absolute true ↑	Absolute false ↓
MHSA + CB	Training	0.636 ± 0.015	0.626 ± 0.012	0.597 ± 0.014	0.535 ± 0.015	0.044 ± 0.002
	Test	0.631	0.622	0.587	0.521	0.040
BiLSTM + MHSA + CB	Training	0.616 ± 0.016	0.614 ± 0.015	0.578 ± 0.016	0.511 ± 0.016	0.047 ± 0.002
	Test	0.629	0.615	0.581	0.514	0.040
TextCNN + CB	Training	0.681 ± 0.012	0.670 ± 0.010	0.639 ± 0.011	0.572 ± 0.010	**0.039 ± 0.001**
	Test	0.702	0.677	0.652	0.585	**0.036**
TextCNN + FFB + CB	Training	0.683 ± 0.013	0.672 ± 0.010	0.641 ± 0.011	0.575 ± 0.011	0.040 ± 0.001
	Test	0.713	0.711	0.673	0.604	**0.036**
ETFC	Training	**0.694 ± 0.008**	**0.683 ± 0.005**	**0.655 ± 0.008**	**0.587 ± 0.011**	0.040 ± 0.001
	Test	**0.724**	**0.717**	**0.684**	**0.617**	**0.036**

a The best values are highlighted in bold. MHSA + classification block (CB), bidirectional long short-term memory network (BiLSTM) + MHSA + CB, TextCNN + CB, TextCNN + feed-forward network block (FFB) + CB.

With the performance comparison of these methods on the MFTP dataset, we find that three TextCNN-based models outperform the two MHSA-based models, and the proposed ETFC model has the most excellent performance on the training set: Precision = 0.694, Coverage = 0.683, Accuracy = 0.655, and Absolute true = 0.587. But the Absolute false (0.040) is slightly worse than that of the TC model (0.039). On the test set, ETFC achieves the best performance on all these five metrics. Compared with the other models, the ETFC model takes into account the optimization of the extracted features of the TextCNN block and the addition of the amino acid position codes, these modules might be important for performance improvement.

### 3.2 Ablation analysis

To investigate the importance of each block in the ETFC model, we performed an ablation analysis for this model, and the experimental results are summarized in Table 2. We evaluate the performance of ETFC and its variants on the test set, including:

**Table 2.** The performance of ETFC and their variants on the test set.^a^

Model	Precision ↑	Coverage ↑	Accuracy ↑	Absolute true ↑	Absolute false ↓
ETFC	0.724	0.717	0.684	0.617	0.036
w/o FFB	0.713	0.708	0.671	0.601	0.036
w/o POS	0.714	0.707	0.671	0.599	0.037
w/o TC	**0.614**	**0.612**	**0.571**	**0.498**	**0.040**

a The worst values are highlighted in bold. w/o, without.

- w/o FFB is a variant that does not use the feed-forward network block.
- w/o POS is a variant that does not use the position encoding embedding block.
- w/o TC is a variant that does not use the TextCNN block.

As shown in Table 2, the overall performance of the model decreases after removing any one block. The result indicates that these blocks in the model are all important to the ETFC model. Notably, without the TextCNN block, the model has the greatest performance decrease. Especially on Accuracy and Absolute true, which decrease by 11.3% and 11.9%, respectively. The results state that the TextCNN block is the most important block for the ETFC model. Compared with the embedding block, the TextCNN block can extract more sequence feature information.

### 3.3 Performance comparison of different loss functions

For the MFTP prediction based on FL and DL, we propose MLFDL to overcome the imbalance problem in MLC. FL and DL-based MLFDL could dynamically adjust the loss cost of the foreground probabilities and the background probabilities for the sample labels. To evaluate the effect of MLFDL on MLC, we compare the performance of the ETFC model with different loss functions, including CEL, FL, ASL, DL, and MLFDL. We also use gradient harmonizing mechanism loss (Li *et al.* 2019) and dice coefficient loss (Li *et al.* 2019), but these two loss functions could not make the model converge. The mathematical expressions of these loss functions are exhibited in Supplementary Table S3, and the performance of our proposed method with different loss functions is shown in Table 3. The results state that the ETFC model with MLFDL achieves the best performance in all measurements.

**Table 3.** The performance comparison of ETFC with different loss functions on the test set.^a^

Loss	Precision ↑	Coverage ↑	Accuracy ↑	Absolute true ↑	Absolute false ↓
CEL	0.709	0.706	0.668	0.600	0.037
FL	0.705	0.693	0.656	0.581	0.038
ASL	0.715	0.698	0.660	0.580	0.039
DL	0.686	0.699	0.658	0.596	**0.036**
MLFDL	**0.724**	**0.717**	**0.684**	**0.617**	**0.036**

a The best values are highlighted in bold.

### 3.4 Performance comparison of ETFC with the existing methods

To further demonstrate the power of the ETFC model, we compare it with the existing methods. To improve the reproducibility and reliability of MPMABP (Li *et al.* 2022), MLBP (Tang *et al.* 2022), sequential properties-recurrent neural network (SP-RNN) (Otović *et al.* 2022) and PrMFTP (Yan *et al.* 2022), we provide the hyperparameter details of these models in Supplementary Tables S4–S7, respectively. The comparison of ETFC with MPMABP, MLBP, SP-RNN, and PrMFTP is performed on the test set, and we randomly selected 80% of the set as the subset and repeated this process five times to obtain five subsets. On each subset, we obtain five metric values for each model. Then, the average of the performance on these subsets is regarded as the final result for each model. To avoid the random sampling error increasing, the Student’s *t*-test with Bonferroni correction (lowering the significance level from 0.01 to 0.002, achieved by dividing 0.01 by 5, Supplementary methods 1.2) is used to determine whether ETFC is significantly different from the other models on the performance metrics. Based on five values for each metric of each model on five test subsets (Supplementary Table S8), we calculate the *P*-values of ETFC and other models for each metric (Supplementary Table S9). As shown in Table 4, ETFC is significantly better than MPMABP, MLBP, SP-RNN, and PrMFTP on the metrics of Precision, Coverage, Accuracy, and Absolute true. Since there are eight shared classes of peptides between ETFC (21 classes of peptides) and MultiPep (20 classes of peptides) (Grønning *et al.* 2021). For a fair comparison, we compare ETFC and MultiPep (https://agbg.shinyapps.io/MultiPep/) on another test set. The dataset process method is used on another test set, to get five another test subsets. On another test subset, our proposed method is also significantly better than MultiPep on Precision, Accuracy, Absolute true and Absolute false (Supplementary Tables S9–S11). In conclusion, the ETFC model has excellent performance for MFTP prediction.

**Table 4.** The performance comparison of ETFC with the state-of-the-art methods on the test set.^a^

Model	Precision ↑	Coverage ↑	Accuracy ↑	Absolute true ↑	Absolute false ↓
MPMABP	0.477*	0.444*	0.430*	0.381*	0.041*
MLBP	0.549*	0.498*	0.493*	0.446*	0.037
SP-RNN	0.604*	0.620*	0.566*	0.482*	0.038*
PrMFTP	0.699*	0.669*	0.651*	0.593*	**0.031** *
ETFC	**0.724**	**0.717**	**0.684**	**0.617**	0.036

a The best values are highlighted in bold. For a fair comparison, each model has been performed with hyperparameter optimization.

* There is significant difference between ETFC and the state-of-the-art method with *P*-value <.002 (the Student’s *t*-test with Bonferroni correction).

### 3.5 Interpretable analysis for ETFC

So far, we just focus on the performance of the ETFC model in terms of MLC, but lack insights into the driving features behind the method. In the proposed ETFC model, the sequence features are extracted by TextCNN block, so the AA information (semantics and position) in the peptide sequence is inevitably disrupted during feature extraction. To gain an interpretable analysis of the ETFC model, we construct a student model with KD to capture the key AAs in the peptide sequences that are important to the prediction results.

#### 3.5.1 Performance of student model in knowledge distillation

In this work, the teacher model is the ETFC model, and the student model is a simple and interpretable model consisting of the backbone block and the classification block. During sequence information extraction in the student model, the dimension of the peptide sequence size is kept constant, and the AA position can be obtained from the feature matrix. Moreover, to make the student model reflect the decision basis of the ETFC model, knowledge is transferred from the teacher model to the student model through KD. Table 5 shows the performance of the student model without KD (Student w/o KD) and the student with KD (Student w/KD) on the test set.

The results show that the Student w/KD model improves the performance on all these MLC metrics. Compared with Student w/o KD, Student w/KD improves Accuracy and Absolute true by 1.2% and 1.6%, respectively. The performance improvement indicates that knowledge is successfully transferred from the teacher model to the student model.

**Table 5.** The performance comparison of the ETFC model with student models on the test set.^a^

Model	Precision ↑	Coverage ↑	Accuracy ↑	Absolute true ↑	Absolute false ↓
Student w/o KD	0.689	0.688	0.647	0.576	0.038
Student w/KD	0.698	0.695	0.659	0.592	**0.036**
ETFC	**0.724**	**0.717**	**0.684**	**0.617**	**0.036**

a The best values are highlighted in bold. “w/” and “w/o” indicate “with” and “without”, respectively.

The Python package *thop* (https://github.com/Lyken17/pytorch-OpCounter) is used to calculate the time complexity (floating point operations per second, FLOPs) and space complexity (the number of parameters in the model) of ETFC and student model w/KD, and the results are listed in Supplementary Table S12. It states that the student model w/KD outperforms ETFC on the time complexity and space complexity, but ETFC has better predictive performance. As stated above, the student model w/KD could be used to interpret the decision basis of the ETFC model.

#### 3.5.2 Interpretation based on student model

To improve the reliability and transparency of decision-making in the model and reveal the role of the attention mechanism in the student model, we visualize the attention weight of each AA corresponding to each function of the peptide sequence and analyze the significant sequence fragments obtained from the model.

**Figure 3:**
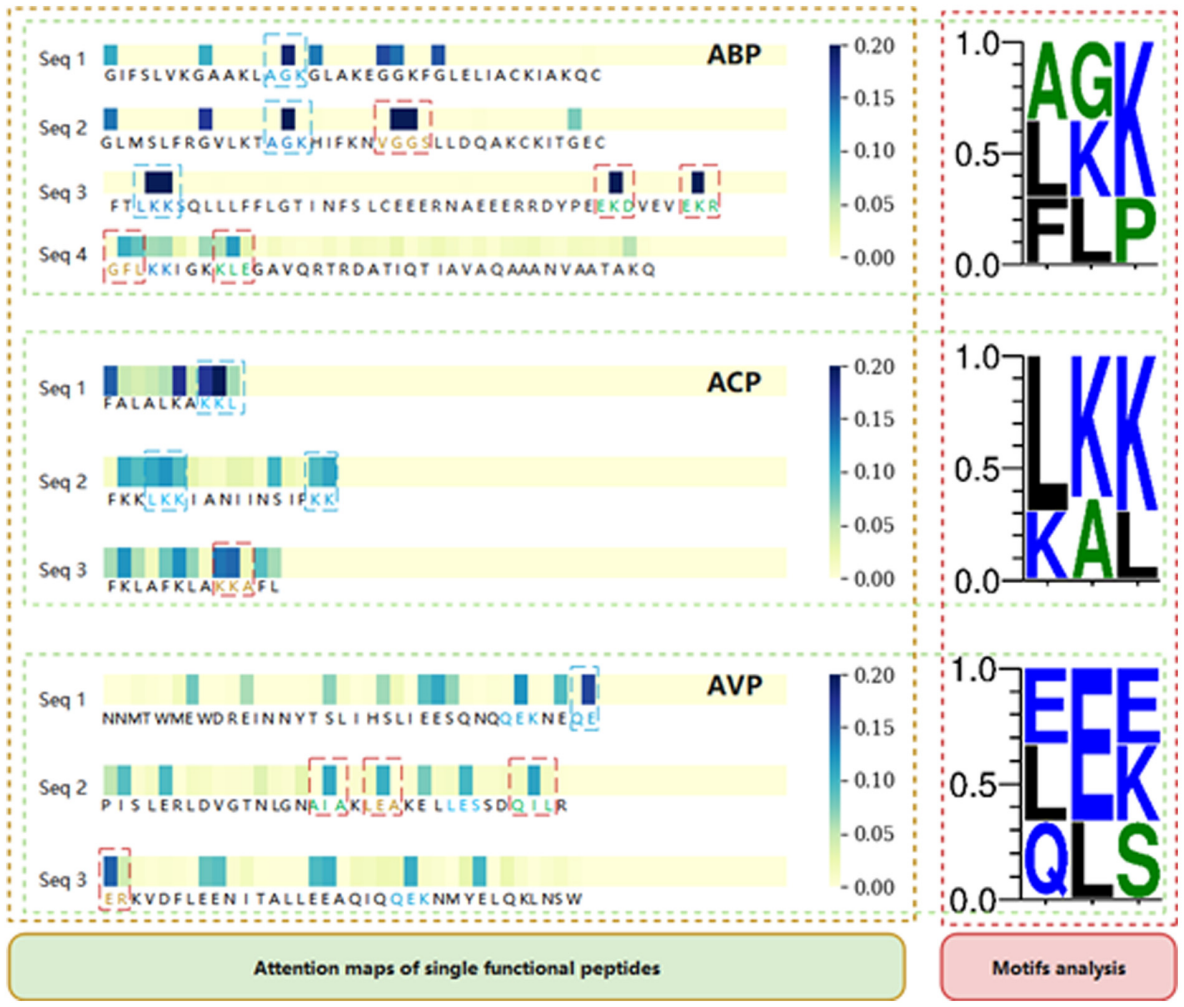
Attention maps and motifs of the peptide sequences with single function. In the motifs analysis on the right, the size of the AA letter indicates the frequency of this AA at that position, with larger sizes indicating larger frequency

Figure 3 shows the attention weights of AA in the sequence for one class of peptides, as well as the underlying motifs in this class of peptides. In the attention weight maps, the attention score is reflected by the color of the AA regions. And the higher the attention scores, the bluer the AA-corresponding regions. We analyze the corresponding functional peptide sequence datasets and obtain sequence motifs that occur frequently in the corresponding functional peptide sequences. To facilitate analyzing the corresponding motifs, we frame the sequence fragment, which contains AA with the largest attention weight and the residue around this AA in the peptide sequence. For the frame with blue, the sequence fragments could be mapped into the motifs. If the frame is red, the motifs do not map any sequence fragments. Taking ABP as an example, for ABP-Seq1 and ABP-Seq2, the “G” has the largest attention weight, and the sequence fragment in the blue frame can correspond to the motif of “AGK.” For ABP-Seq3, the “K” has the largest attention weight, and the sequence fragment framed in blue can also correspond to the motif of “LKK.” This indicates that our model can learn critical sequence information in peptide sequences. In addition, we note that the sequence segments in the red frames in ABP-Seq2, Seq3, and Seq4 do not correspond exactly to the motifs, e.g. “VGGS” in the red frame in ABP-Seq2, “EKD” and “EKR” in the red frame in ABP-Seq3. Though these sequence fragments are not mapped to any motif, they may be the unique important region for peptides to be identified as one function class. It suggests that our model can discover potential sequence patterns that cannot be found by simple analysis of datasets, especially for datasets that do not include sequences of sequential motif peptides.

**Figure 4:**
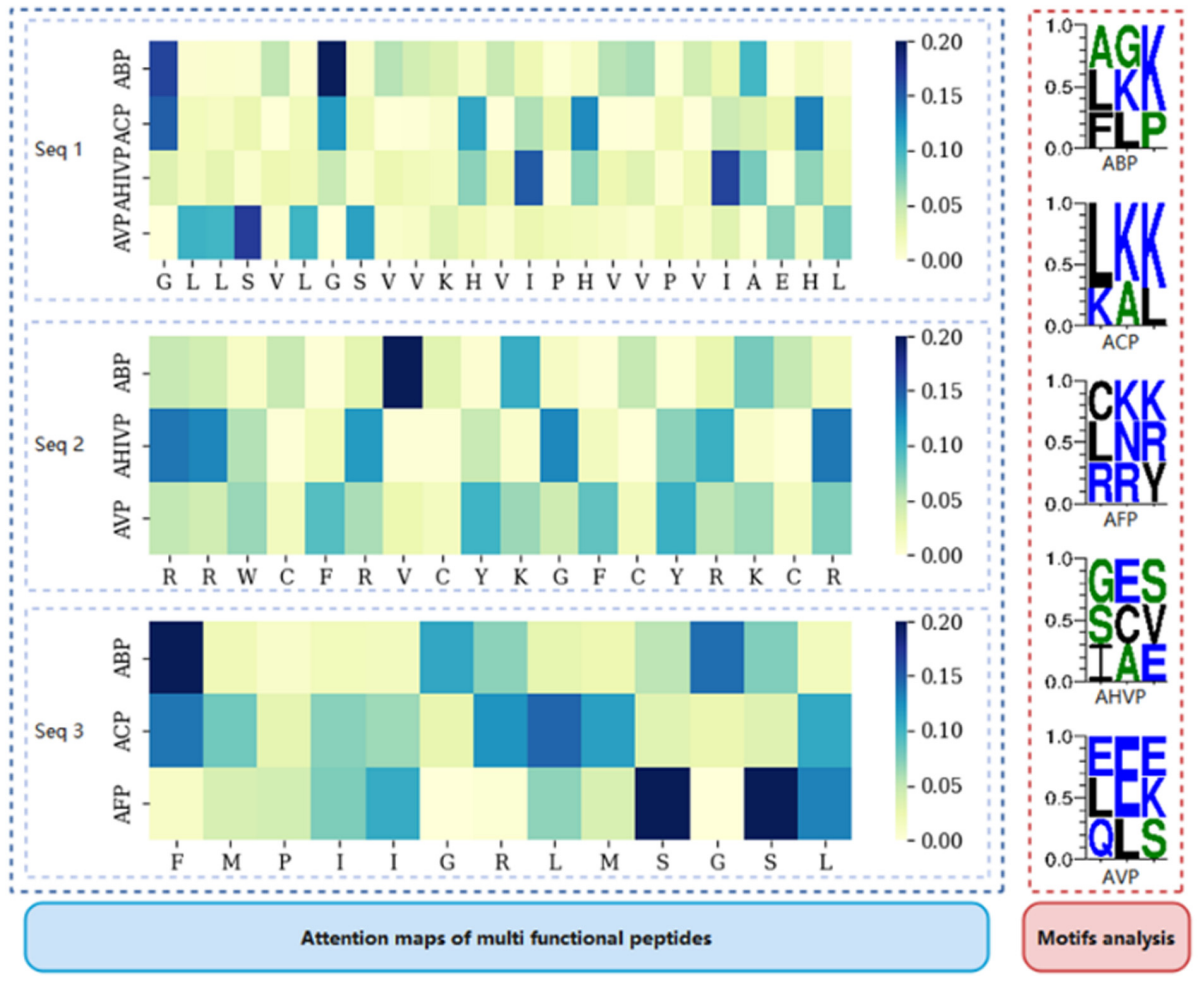
Attention maps and motifs of the peptide sequences with multifunctions

To illustrate the attention weight of AA in the MFTP, we visualized attention weight maps for three peptide sequences with multiple functions, as shown in Fig. 4. For these peptide sequences, our model focuses on different AAs for different functions, especially since these sequence segments are difficult to match with motifs. This indicates that our model can adaptively extract sequence information for therapeutic peptides with different functions.

**Figure 5:**
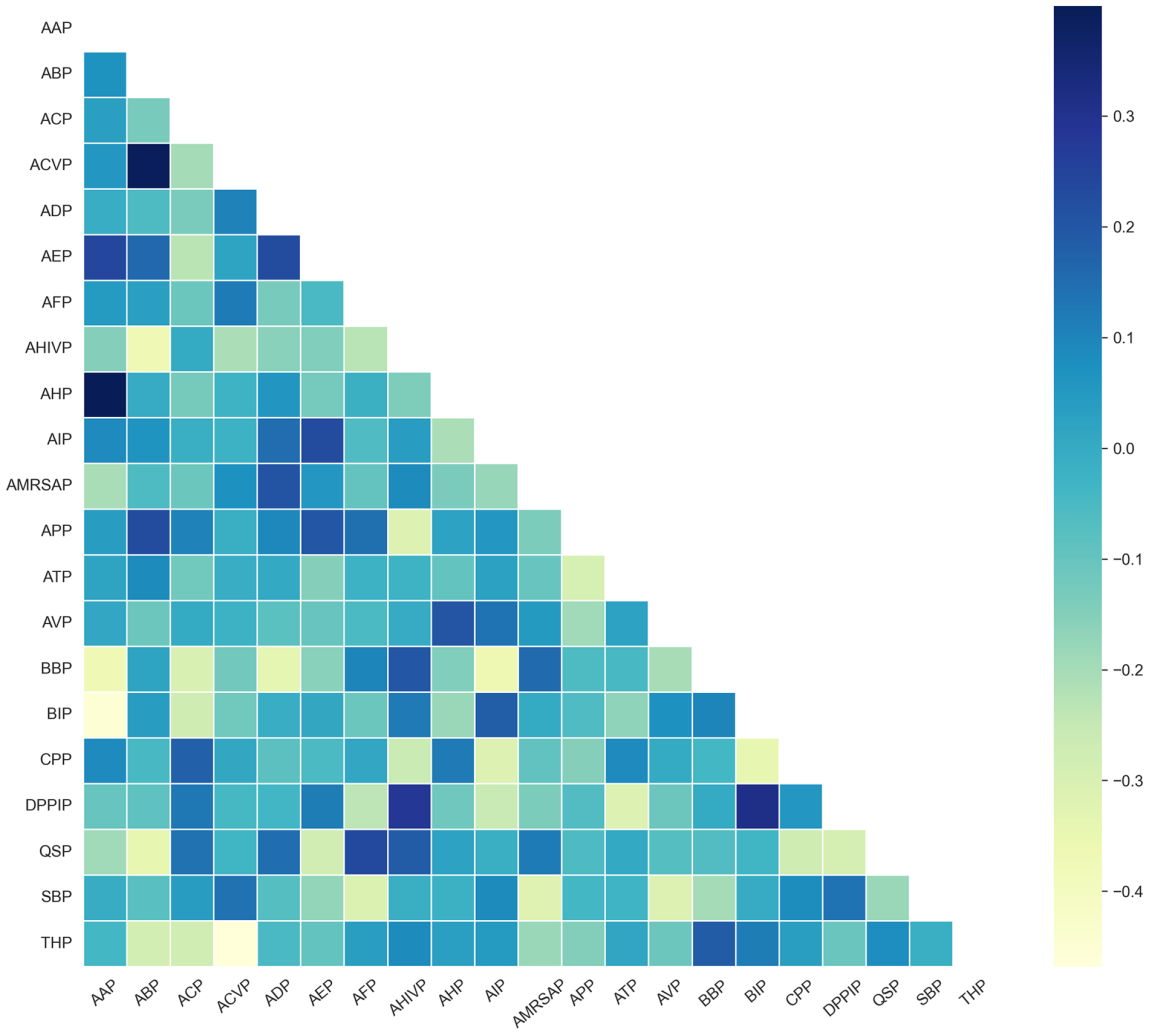
Association between the weight vector of the 21 categories of therapeutic peptides revealed by our model

In addition, we extract the weights of the feed-forward neural network within the masked self-attention mechanism of the student model. These weights correspond to 21 vector representations of the 21 functions. We then calculate the Pearson correlation coefficient between the pairwise vector representations to reveal the similarity of the model’s attention to individual AAs in the same peptide sequence with different functions (Fig. 5). There is very little correlation between the pairwise vectors, which is highly consistent with what we observe in Fig. 4. The result suggests that the AA in the peptide sequences plays different roles on function identification.

## 4 Conclusion

In this work, the ETFC model is proposed to predict MFTP. Compared with the existing multi-label methods, ETFC achieves the best performance. MLFDL is applied in the ETFC model to solve the inherent imbalance problem in the multi-label dataset and achieve competitive performance. In addition, with the teacher–student framework-based KD, we track the contribution of each AA in the peptide sequence for each class and enhance the interpretability of the model. It is anticipated that predictor ETFC will become a very useful high-throughput tool for identifying MFTP and assist biologists to screen potential peptide drugs efficiently.

## Supplementary Material

btad334_Supplementary_Data
