# Supplementary material for: Deep learning-based multi-functional therapeutic peptides prediction with a multi-label focal dice loss function
Source: Bioinformatics. 2023 May 22;39(6):btad334. doi: 10.1093/bioinformatics/btad334 (PMC10234765; doi:10.1093/bioinformatics/btad334)
Supplement: btad334_Supplementary_Data [file btad334_supplementary_data.docx]

# Supplementary Information:

# Deep learning-based multi-functional therapeutic peptides prediction with a multi-label focal dice loss function

Henghui Fan, Wenhui Yan, Lihua Wang, Jie Liu, Yannan Bin* and Junfeng Xia*

Key Laboratory of Intelligent Computing and Signal Processing of Ministry of Education and Information Materials and Intelligent Sensing Laboratory of Anhui Province, Institutes of Physical Science and Information Technology, Anhui University, Hefei, Anhui 230601, China.

*Corresponding authors: Yannan Bin, E-mail: ynbin@ahu.edu.cn; Junfeng Xia, E-mail: jfxia@ahu.edu.cn.

1. **Supplementary methods**

**1.1 Student model**

The student model is mainly composed of a backbone block and a classification block. With the same data preprocessing used in the ETFC model, the input peptide sequences are imported into the backbone block, and the output is fed into the classification block. Finally, the corresponding prediction scores are obtained.

The backbone block is composed of masked multi-head self-attention mechanism (MMHSA)(Nicolson and Paliwal, 2020), residual connection, layer normalization, and masked self-attention mechanism (MSA). This block is used to extract the correlation information between the AAs in the peptide sequence, and transform the representation of the AA feature vector by MSA. With this block, the AAs in the peptide sequence can be given the various contribution weights corresponding to 21 different functions. The classification block is employed to predict the function of the peptide sequence by the full connection layers.

MSA is used to track the contribution of each AA in peptide sequences and avoid the interference of the zero-padding method. The mathematical expression of MSA is described as follows:

$$\left\{ \begin{matrix} Q=X\cdot W_{q} \\ K=X\cdot W_{k} \\ V=X\cdot W_{v} \end{matrix} \right. (1)$$

$$Masked\_self\_attention(Q,K,V)=masked\_softmax(W_{a}\cdot\frac{QK^{T}}{\sqrt{d_{k}}})\cdot V (2)$$

where $X\in\mathbb{R}^{L\times d_{m}}$ is the output of MMHSA. Through the linear layers $W_{q}$, $W_{k}$, and $W_{v}\in\mathbb{R}^{d_{m}\times d_{k}}$, *X* is transformed to query matrix $Q\in\mathbb{R}^{L\times d_{k}}$, key matrix $K\in\mathbb{R}^{L\times d_{k}}$, and value matrix $V\in\mathbb{R}^{L\times d_{k}}$, respectively. After getting the attention score by scaled dot-product attention, the score matrix is transformed to attention weight through the linear layer $W_{a}\in\mathbb{R}^{N\times L}$. Here, *L* is the length of the input sequence, *N* is the number of labels, $d_{m}$ is the embedding dimension and $d_{k}$ is the dimension of matrix Q, K, and V.

**1.2 Statistical test**

In this work, we obtain the results for each metrics of each model (Precision, Coverage, Accuracy, Absolute true, Absolute false) on five test subsets and another test subsets. These results are used to calculate the test statistics and p-values of ETFC and other models on each performance metric. See Table S4 and S6 for the results of each model on each test subset and another test subset. To avoid the random sampling error increasing, the Student’s t-test with Bonferroni correction (lowering the significance level from 0.01 to 0.002, achieved by dividing 0.01 by 5) is used to determine whether ETFC is significantly different from the the state-of-the-art models (MultiPep(Grønning, et al., 2021), MPMABP (Li, et al., 2022), MLBP(Tang, et al., 2022), SP-RNN (Otović, et al., 2022) and PrMFTP (Yan, et al., 2022)) on the five performance metrics (Precision, Coverage, Accuracy, Absolute true, Absolute false). If the null hypothesis is rejected (p-value < 0.002), we know that the metrics of ETFC are significantly different from those of the state-of-the-art models.

**References**

Grønning, A.G. *et al.* (2021) MultiPep: a hierarchical deep learning approach for multi-label classification of peptide bioactivities. *Biology Methods and Protocols*;6(1):bpab021.

Li, Y. *et al.* (2022) MPMABP: a CNN and Bi-LSTM-Based method for predicting multi-activities of bioactive peptides. *Pharmaceuticals*;15(6):707.

Nicolson, A. and Paliwal, K.K. (2020) Masked multi-head self-attention for causal speech enhancement. *Speech Commun*;125:80-96.

Otović, E. *et al.* (2022) Sequential Properties Representation Scheme for Recurrent Neural Network-Based Prediction of Therapeutic Peptides. *Journal of Chemical Information and Modeling*;62(12):2961-2972.

Tang, W. *et al.* (2022) Identifying multi-functional bioactive peptide functions using multi-label deep learning. *Briefings Bioinf*;23(1):bbab414.

Yan, W. *et al.* (2022) PrMFTP: multi-functional therapeutic peptides prediction based on multi-head self-attention mechanism and class weight optimization. *PLoS Comput Biol*;18(9):e1010511.

1. **Supplementary figures**


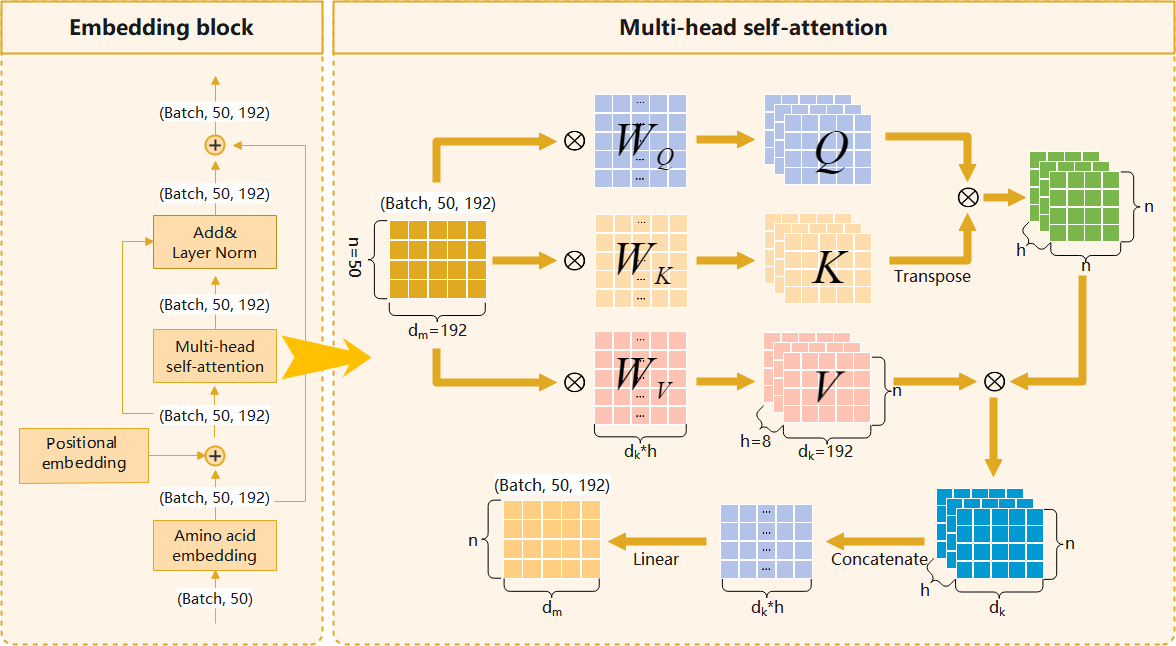


Fig. S1. The framework of the embedding block and the multi-head self-attention. *n*, the sequence length. *d_m_* and *d_k_*, the dimensions of the embedding and hidden layer. *h*, the number of heads.


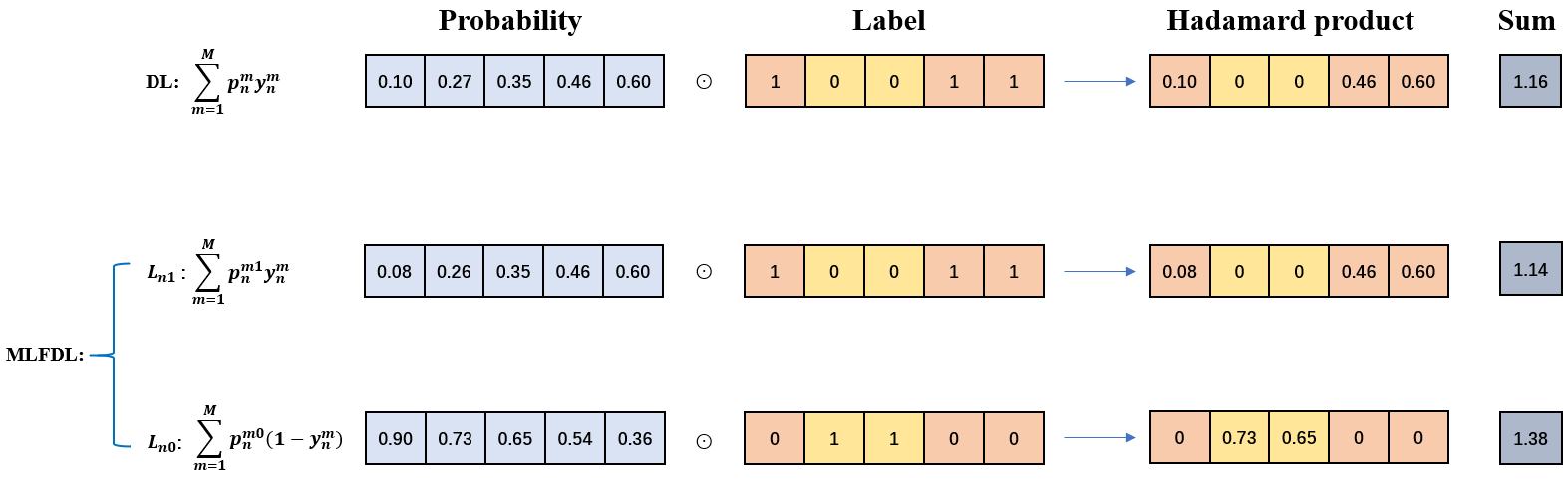
Fig. S2. Simple examples of calculation procedures for the molecular parts of DL and MLFDL. It is assumed that the hyperparameters are $\varepsilon_{1}=0.7$ and $\varepsilon_{0}=0.5$.

1. **Supplementary tables**

Table S1. The performance comparison of five-fold cross-validation results of ETFC under different cut-off thresholds on the training set.

| **Threshold** | **Precision ↑** | **Coverage ↑** | **Accuracy ↑** | **Absolute true↑** | **Absolute false ↓** |
| --- | --- | --- | --- | --- | --- |
| 0.4 | 0.689 | 0.679 | 0.648 | 0.585 | **0.040** |
| 0.5 | **0.694** | **0.683** | **0.655** | **0.587** | **0.040** |
| 0.6 | 0.690 | 0.682 | 0.650 | 0.581 | 0.041 |

*Note:* The best values are highlighted in bold.

**Table S2. The parameter details of the ETFC model.**

| **Module** | **Parameter** | **Scope** | **Optimal** |
| --- | --- | --- | --- |
| Embedding block | Embedding dimension | [64, 100, 128, 192] | 192 |
|  | Attention head Size | [4, 8] | 8 |
| TextCNN block | Pooling size | [4, 5, 6] | 5 |
| Training module | Batch size | [64, 128, 192, 256] | 192 |
|  | Epochs | [200, 250] | 200 |
|  | Learning rate | (0.001, 0.01) | 0.0018 |
| Multi-label focal dice loss | $\varepsilon_{1}$ | [0, 0.3, 0.5, 0.7, 1.0] | 0.7 |
|  | $\varepsilon_{0}$ | [0, 0.3, 0.5, 0.7, 1.0] | 0.5 |
|  | $\omega$ | [0, 0.3, 0.5, 0.7, 1.0] | 0.3 |

*Notes:* [ ] indicates that one value is selected from the given values, and ( ) indicates that the value is taken from the given value range.

Table S3. The mathematical expressions of different loss functions.

| **Loss** | **Formula (for one sample** $\boldsymbol{x}_{\boldsymbol{n}}$**)** |
| --- | --- |
| CEL | $-\sum_{m=1}^{M} \left[ y_{n}^{m}logp_{n}^{m}+(1-y_{n}^{m})log{(1-p}_{n}^{m}) \right]$ |
| FL | $-\alpha_{n}\sum_{m=1}^{M} \left[ \left( 1-p_{n}^{m} \right)^{\gamma}y_{n}^{m}logp_{n}^{m}+\left( p_{n}^{m} \right)^{\gamma}(1-y_{n}^{m})log{(1-p}_{n}^{m}) \right]$ |
| ASL | $-\sum_{m=1}^{M} \left[ \left( 1-p_{n}^{m} \right)^{\gamma+}y_{n}^{m}logp_{n}^{m}+\left( max\left( p_{n}^{m}-\tau, 0 \right) \right)^{\gamma-}(1-y_{n}^{m})log(1-max\left( p_{n}^{m}-\tau, 0 \right)) \right]$ |
| DL | $1-\frac{2\sum_{m=1}^{M} p_{n}^{m}y_{n}^{m}+\delta}{\sum_{m=1}^{M} \left( p_{n}^{m} \right)^{2}+\sum_{m=1}^{M} \left( y_{n}^{m} \right)^{2}+\delta}$ |
| MLFDL | $\omega\left[ 1-\frac{2\sum_{m=1}^{M} p_{n}^{m}y_{n}^{m}}{\sum_{m=1}^{M} \left( p_{n}^{m1} \right)^{2}+\sum_{m=1}^{M} \left( y_{n}^{m} \right)^{2}} \right]+(1-\omega)\left[ 1-\frac{2\sum_{m=1}^{M} p_{n}^{m0}(1-y_{n}^{m})}{\sum_{m=1}^{M} \left( p_{n}^{m0} \right)^{2}+\sum_{m=1}^{M} \left( 1-y_{n}^{m} \right)^{2}} \right]$ |

*Notes*: $x_{n}$ is the *n*-th sample. *M* represents the number of labels in the sample. CEL, cross-entropy loss. FL, focal loss. ASL, asymmetric loss. DL, dice loss. MLFDL, multi-label focal dice loss.

**Table S4. The parameter details of the MPMABP model.**

| **Module** | **Parameter** | **Scope** | **Optimal** |
| --- | --- | --- | --- |
| Embedding | Embedding dimension | [100, 150] | 100 |
| Pooling | Pooling size | [3, 5] | 3 |
| Training module | Learning rate | [0.001, 0.002] | 0.002 |
|  | Dropout | [0.5, 0.6] | 0.5 |

*Note:* [ ] indicates that one value is selected from the given values.

**Table S5. The parameter details of the MLBP model.**

| **Module** | **Parameter** | **Scope** | **Optimal** |
| --- | --- | --- | --- |
| Embedding | Embedding dimension | [100, 150] | 100 |
| Pooling | Pooling size | [3, 5] | 5 |
| Training module | Learning rate | [0.001, 0.002] | 0.001 |
|  | Dropout | [0.5, 0.6] | 0.5 |

*Note:* [ ] indicates that one value is selected from the given values.

**Table S6. The parameter details of the SP-RNN model.**

| **Module** | **Parameter** | **Scope** | **Optimal** |
| --- | --- | --- | --- |
| Embedding | Embedding dimension | [100, 150] | 100 |
| CNN layer1 | Kernel size | [32, 64] | 64 |
| CNN layer2 | Kernel size | [32, 64] | 64 |
| CNN layer3 | Kernel size | [4, 6] | 6 |
| LSTM | Number of units | [64, 128] | 64 |
| Training module | Dropout | [0.1, 0.2, 0.3] | 0.1 |

*Note:* [ ] indicates that one value is selected from the given values.

**Table S7. The parameter details of the PrMFTP model.**

| **Module** | **Parameter** | **Scope** | **Optimal** |
| --- | --- | --- | --- |
| Embedding | Embedding dimension | [64, 128, 192] | 128 |
| Pooling | Pooling size | [3, 5] | 5 |
| BiLSTM | Number of units | [50, 100, 150] | 100 |
| Multi-head self-attention | Attention head size | [5, 8] | 5 |
| Training module | Learning rate | [0.001, 0.002] | 0.001 |
|  | Dropout | [0.5, 0.6] | 0.6 |

*Note:* [ ] indicates that one value is selected from the given values.

**Table S8. Performance of MPMABP, MLBP, SP-RNN, PrMFTP and ETFC on five test subsets.**

| **(A) Test subset_1** | | | | | |
| --- | --- | --- | --- | --- | --- |
| **Model** | **Precision↑** | **Coverage↑** | **Accuracy↑** | **Absolute true↑** | **Absolute false↓** |
| MPMABP | 0.480 | 0.478 | 0.446 | 0.386 | 0.040 |
| MLBP | 0.545 | 0.495 | 0.489 | 0.441 | 0.038 |
| SP-RNN | 0.600 | 0.618 | 0.563 | 0.480 | 0.038 |
| PrMFTP | 0.697 | 0.663 | 0.646 | 0.586 | **0.032** |
| ETFC | **0.724** | **0.717** | **0.684** | **0.618** | 0.036 |
| **(B) Test subset_2** | | | | | |
| MPMABP | 0.479 | 0.436 | 0.428 | 0.381 | 0.042 |
| MLBP | 0.550 | 0.500 | 0.496 | 0.450 | 0.037 |
| SP-RNN | 0.600 | 0.616 | 0.563 | 0.480 | 0.038 |
| PrMFTP | 0.703 | 0.670 | 0.654 | 0.598 | **0.030** |
| ETFC | **0.724** | **0.718** | **0.684** | **0.617** | 0.036 |
| **(C) Test subset_3** | | | | | |
| MPMABP | 0.475 | 0.432 | 0.424 | 0.379 | 0.042 |
| MLBP | 0.545 | 0.495 | 0.491 | 0.446 | 0.037 |
| SP-RNN | 0.606 | 0.622 | 0.568 | 0.484 | 0.038 |
| PrMFTP | 0.705 | 0.673 | 0.654 | 0.594 | **0.031** |
| ETFC | **0.724** | **0.717** | **0.683** | **0.617** | 0.036 |
| **(D) Test subset_4** | | | | | |
| MPMABP | 0.474 | 0.434 | 0.424 | 0.378 | 0.042 |
| MLBP | 0.550 | 0.498 | 0.492 | 0.443 | 0.037 |
| SP-RNN | 0.604 | 0.619 | 0.565 | 0.480 | 0.039 |
| PrMFTP | 0.695 | 0.668 | 0.649 | 0.593 | **0.031** |
| ETFC | **0.725** | **0.717** | **0.685** | **0.618** | 0.036 |
| **(E) Test subset_5** | | | | | |
| MPMABP | 0.479 | 0.438 | 0.429 | 0.382 | 0.042 |
| MLBP | 0.554 | 0.504 | 0.499 | 0.451 | 0.037 |
| SP-RNN | 0.608 | 0.624 | 0.571 | 0.486 | 0.038 |
| PrMFTP | 0.698 | 0.671 | 0.652 | 0.594 | **0.031** |
| ETFC | **0.722** | **0.718** | **0.682** | **0.615** | 0.036 |

*Note:* The best values are highlighted in bold on each test subset.

Table S9. P-values from the Student’s t-test for other model VS ETFC on different metrics.

| **Other model VS ETFC** | **Precision** | **Coverage** | **Accuracy** | **Absolute true** | **Absolute false** |
| --- | --- | --- | --- | --- | --- |
| MPMABP^a^ | 9.064e-9 | 6.180e-6 | 4.032e-7 | 9.735e-9 | 1.510e-4 |
| MultiPep^b^ | 1.155e-7 | 3.232e-5 | 7.999e-8 | 6.908e-8 | 1.037e-7 |
| MLBP^a^ | 1.074e-7 | 1.262e-8 | 9.442e-8 | 2.401e-7 | 4.636e-3 |
| SP-RNN^a^ | 3.802e-7 | 2.802e-7 | 4.910e-7 | 1.734e-7 | 3.882e-4 |
| PrMFTP^a^ | 2.598e-4 | 7.678e-6 | 5.873e-5 | 4.271e-4 | 9.349e-5 |

*Notes:* ^a^ Student’s t-test is performed on the test set. ^b^ Student’s t-test is performed on another test set. The significance level with Bonferroni correction is 0.002.

**Table S10**. **Performance of MultiPep and ETFC on five another test subsets.**

| **(A) Another test subset_1** | | | | | |
| --- | --- | --- | --- | --- | --- |
| **Model** | **Precision↑** | **Coverage↑** | **Accuracy↑** | **Absolute true↑** | **Absolute false↓** |
| MultiPep | 0.426 | **0.743** | 0.412 | 0.164 | 0.079 |
| ETFC | **0.664** | 0.677 | **0.628** | **0.547** | **0.040** |
| **(B) Another test subset_2** | | | | | |
| MultiPep | 0.430 | **0.743** | 0.417 | 0.172 | 0.079 |
| ETFC | **0.680** | 0.692 | **0.638** | **0.548** | **0.039** |
| **(C) Another test subset_3** | | | | | |
| MultiPep | 0.425 | **0.744** | 0.411 | 0.161 | 0.079 |
| ETFC | **0.677** | 0.692 | **0.639** | **0.555** | **0.039** |
| **(D) Another test subset_4** | | | | | |
| MultiPep | 0.427 | **0.741** | 0.414 | 0.167 | 0.078 |
| ETFC | **0.670** | 0.681 | **0.629** | **0.542** | **0.040** |
| **(E) Another test subset_5** | | | | | |
| MultiPep | 0.426 | **0.738** | 0.413 | 0.169 | 0.078 |
| ETFC | **0.669** | 0.682 | **0.629** | **0.541** | **0.040** |

*Note:* The best values are highlighted in bold on each another test subset.

Table S11. The performance comparison of ETFC with MultiPep on another test set with eight shared classes.

| **Model** | **Precision ↑** | **Coverage ↑** | **Accuracy ↑** | **Absolute true ↑** | **Absolute false ↓** | |
| --- | --- | --- | --- | --- | --- | --- |
| MultiPep | 0.426* | **0.742*** | 0.413* | 0.167* | 0.079* |  |
| ETFC | **0.672** | 0.685 | **0.633** | **0.547** | **0.040** |  |

*Notes:* The best value is highlighted in bold. *means that ETFC is significantly different from MultiPep with p-value < 0.002 (the Student’s t-test with Bonferroni correction).

Table S12. Comparison of the complexity and performance between ETFC and student model with KD.

| Model | FLOPs ↓ | Parameters ↓ | Accuracy ↑ | Absolute true ↑ |
| --- | --- | --- | --- | --- |
| Student w/ KD | 3.424×10^9^ | 1.236×10^7^ | 0.659 | 0.592 |
| ETFC | 6.209×10^9^ | 2.439×10^7^ | 0.684 | 0.617 |
